# Tumor Cell Dormancy: Threat or Opportunity in the Fight against Cancer

**DOI:** 10.3390/cancers11081207

**Published:** 2019-08-19

**Authors:** Rana Jahanban-Esfahlan, Khaled Seidi, Masoud H. Manjili, Ali Jahanban-Esfahlan, Tahereh Javaheri, Peyman Zare

**Affiliations:** 1 Drug Applied Research Center, Tabriz University of Medical Sciences, Tabriz 9841, Iran; 2 Department of Medical Biotechnology, Faculty of Advanced Medical Sciences, Tabriz University of Medical Sciences, Tabriz 9841, Iran; 3 Student Research Committee, Tabriz University of Medical Sciences, Tabriz 9841, Iran; 4 Biotechnology Research Center, Tabriz University of Medical Sciences, Tabriz 9841, Iran; 5 Department of Microbiology & Immunology, VCU School of Medicine, Massey Cancer Center, Richmond, VA 23298, USA; 6 Stem Cell Research Center, Tabriz University of Medical Sciences, Tabriz 9841, Iran; 7 Ludwig Boltzmann Institute for Cancer Research, 1090 Vienna, Austria; 8 Faculty of Medicine, Cardinal Stefan Wyszyński University in Warsaw, 01-938 Warsaw, Poland

**Keywords:** tumor dormancy, tumor relapse, tumor escape, metastasis, cancer therapy

## Abstract

Tumor dormancy, a clinically undetectable state of cancer, makes a major contribution to the development of multidrug resistance (MDR), minimum residual disease (MRD), tumor outgrowth, cancer relapse, and metastasis. Despite its high incidence, the whole picture of dormancy-regulated molecular programs is far from clear. That is, it is unknown when and which dormant cells will resume proliferation causing late relapse, and which will remain asymptomatic and harmless to their hosts. Thus, identification of dormancy-related culprits and understanding their roles can help predict cancer prognosis and may increase the probability of timely therapeutic intervention for the desired outcome. Here, we provide a comprehensive review of the dormancy-dictated molecular mechanisms, including angiogenic switch, immune escape, cancer stem cells, extracellular matrix (ECM) remodeling, metabolic reprogramming, miRNAs, epigenetic modifications, and stress-induced p38 signaling pathways. Further, we analyze the possibility of leveraging these dormancy-related molecular cues to outmaneuver cancer and discuss the implications of such approaches in cancer treatment.

## 1. Introduction

Microorganisms adopt different mechanisms to survive under hostile conditions. They undergo drastic changes in cellular physiology to shape the surrounding microenvironment to best fit their requirements [1]. In response to a stressor such as chemotherapy, radiation therapy, or O_2_/nutrient scarcity, stressed tumor cells that survive apoptosis become dormant. After cessation of the therapy, the dormant cells may repopulate, resulting in tumor recurrence and development of chemotherapy-resistant cancer cells [2,3].

Dormant cells can be detected as circulating tumor cells (CTCs) in the bloodstream or disseminated cells (DTCs) in secondary sites such as bone marrow (BM). Once metastatic cells find a new home to settle, they may undergo different fates: they either die, remain silent (restrictive soil), or grow with an even more aggressive and lethal behavior than before (permissive soil). The implication of tumor cell dormancy is very well established in the development of multidrug resistance (MDR), minimum residual disease (MRD), tumor outgrowth, and metastatic relapse, leading to cancer treatment failure [4,5,6].

In the context of clinical settings, clinical dormancy is defined as the pre- or post-treatment time point where residual cancer cells are clinically undetectable. Generally, cancer dormancy can be categorized as cellular dormancy or tumor mass dormancy [7]. Cellular dormancy is defined as transient G0-G1 growth arrest in a certain fraction of cancer cells (dormant cells); accordingly, cancer progression occurs when a single dormant malignant cell gains the ability to re-enter the cell cycle. In tumor mass dormancy a stagnation of total tumor growth due to the equilibrium of proliferation and apoptosis is governed by an angiogenic switch or immune escape (two prevailing mechanisms) that eventually shifts the balance in favor of cancer progression [8]. Accordingly, current in vitro, in vivo and ex vivo (organs-on-a-chip) experimental models of cancer dormancy can mimic cellular and tumor mass dormancy (angiogenic and immunologic dormancy), each reflecting distinct growth kinetics [9]. These models were somewhat successful in recapitulating dormancy mechanism of tumors, as they lead to the discovery of cellular dormancy factors/mechanisms including extracellular matrix (ECM) [10], metabolic [11,12], epigenetic [13,14], stemness [15,16], non-coding RNAs [17], and p38 stress-induced signaling pathway [18,19,20], as we intend to discuss in detail in this review (Table 1). However, these models fail to address the full picture of clinically observed dormancy-related cellular processes, such that, the function of angiogenic dormancy in metastatic dormancy is unclear [21]. Also, the paradox role of pro- and anti-proliferative action of the immune system is shown in tumor evolution [22]. Also, inherent difficulties in detecting and characterizing micrometastatic lesions/individual cells in the patient is another hurdle to assess the relative contribution of various mechanisms of dormancy in the clinical setting [23]. However, the new emerging methodologies, namely microfluidics based on analyzing microliters of serum samples may enable detection of single cells, as wells as DTCs and may aid to capture dormancy dynamics at a single-cell resolution (reviewed in [24,25,26,27]). A full discussion of dormancy-related experimental models is recently reviewed [28,29,30]. The focus of the current paper, however, is to employ the data extracted from different dormancy models in the preclinical settings to highlight the stealth mechanisms dictating cancer cell dormancy. Subsequently, we analyze the possibility of leveraging dormancy related molecular cues to outmaneuver cancer. Finally, the implication of each approach in cancer treatment and prognosis is discussed.

## 2. Angiogenic Switch

As a foremost contributing factor to tumor dormancy is the incompetence of proliferating tumors to acquire the angiogenic potential to induce the formation of new blood vessels [97]. In the same way, the ability to drive tumor angiogenesis process will promote the escape from latency and initiate tumor outgrowth. This point of cancer progression is recognized as an angiogenic switch, the induction of which is under the control of intricate biological processes comprising the cancer cells, BM-derived endothelial precursors, and tumor associated-stromal microenvironment [98,99].

Angiogenesis switch, or in a better word, angiogenic failure is a key piece of the cancer cell dormancy puzzle. The role of angiogenic dormancy has been highlighted by the experimental as well as clinical observations that anti-angiogenic therapies or angiogenic therapies could induce tumor dormancy or rescue dormant cells, respectively (Table 1). As such, angiogenic factors display a reduced expression in dormant cells; meanwhile, the high transcription level of angiogenesis inhibitors such as angiostatin, endostatin, and thrombospondin-1 is associated with the tumor dormancy.

Concerning the role of the angiogenic switch in cancer dormancy, two therapeutic approaches follow: in the first approach, long-term dormancy can be induced using anti-angiogenic therapy as validated in different angiogenic models of dormancy in vivo [100]. For example, cancer patients may benefit from bioactive angiostatin gene therapy; however, transgene delivery may necessitate repetitive drug administration to assure persistent latency state of primary tumors and control metastatic expansion, that is not clinically feasible because of off-target effects and also low expression level of transgene as shown in a B16F10 melanoma mouse model [31]. Also, eradication of primary tumors which result in inducing dormant tumor cells is evidenced by the high expression level of angiogenic inhibitor thrombospondin-1 in human melanoma xenografts (D-12, R-18, and U-25). In contrast to wild-type tumors with no thrombospondin-1 expression, a tumor-suppressive index was demonstrated only in mice bearing D-12, U-25, or thrombospondin-1 overexpressing R-18 tumors, as validated by melanoma angiogenesis, lung colonization, and spontaneous pulmonary metastasis [32]. These results suggest a combination of sustained anti-angiogenic therapy besides local treatment for melanoma therapy. Impaired tumor angiogenesis also drives prolonged dormancy of human liposarcoma. This type of tumor with no angiogenic activity produce moderately high amount of the anti-angiogenesis agents TIMP-1 and thrombospondin-1, proposing that the nonangiogenic, microscopic, dormant stage of melanoma might be susceptible to anti-angiogenic treatment years prior to manifestation of malignant disease, or macroscopically detection of anatomical site of a given tumor, by current approaches [101]. Notably, metastatic dormancy can be realized once the proliferation of tumor cell is well-balanced with an equal ratio of tumor cell apoptosis, indicating that anti-angiogenic treatment regulates metastatic outgrowth through increasing of cancer cell death [21].

Besides, the dormancy process can be reversed when dormant cells are fed with adequate angiogenic factors. The reversibility of dormancy envisages the second approach based on awakening and switching silent cells into proliferative and yet susceptible cancer cells to further face the anti-angiogenic therapy. In this line, studies on engineered organotypic microvascular niches established that while a Thrombospondin-1 secretion from endothelial cells induces maintained BCC dormancy, bioactive Periostin and TGF-β1 function as tumor-promoting factors in sprouting neovasculature which further sparks micrometastatic outgrowth [35]. A detailed role of TGF-β related cellular dormancy is recently reviewed in [102]. Moreover, the shift of resting tumors was shown to be correlated with the downregulation of Thrombospondin and a reduced response to Angiostatin in angiogenic tumors. The transformation of quiescent tumors to highly proliferating tumors was also associated with the activation and regulation of molecular cues connected to tumor dormancy in different in vivo cancer models [36]. Also, engineered WM1341B cells constitutively overexpressing the vascular endothelial growth factor (VEGF)/vascular permeability factor (VPF) isoform is shown to terminate long-term melanoma dormancy and can induce overt and progressively growing tumors. These effects were largely counteracted by neutralizing VEGF activity in mice [33].

## 3. Cancer Immune-Mediated Dormancy

**Figure 1:**
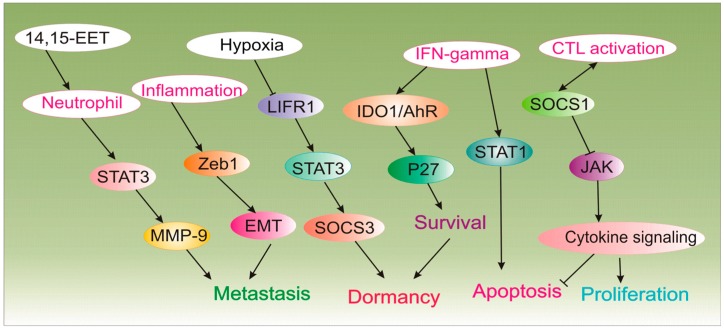
The implication of the immune system in tumor cell dormancy.

The tumor-host interactions are progressively known as crucial constituents of cancer growth or inhibition. Particularly, infiltrating immune cells are critical features of the tumor microenvironment [103]. Cancer cells are encircled by stromal cells, including macrophages, fibroblasts, mast cells, neutrophils, and lymphocytes, which communicate through an intricate system of intercellular signaling pathways, mediated by cytokines, adhesion molecules, and the receptor-ligand network [42]. The immune system can confine the metastatic spread of cancer cells, encouraging their long-lasting latency [104]. Nonetheless, the perturbation of the equilibration in favor of immune escape will result in tumor outgrowth rather than the destruction of cancer cells, otherwise, immune surveillance could maintain angiogenic control and impede cancer cell growth (Figure 1) [105]. In this context, results from a valuable study clearly indicated the immune-mediated dormancy in a nontransgenic mouse model, where injection of MHC1-negative GR9-B11 mouse fibrosarcoma clone results in no apparent spontaneous metastases within 24 months after tumor cell injection receiving no antitumor treatment. Of note, immunodepletion of T lymphocytes or asialo GM1-positive cells results in relieving the restraint dormant cells into disseminated MHC-1 positive metastatic cells in mice lungs [106]. Thus, immune-mediated cancer dormancy at least can be regulated by T lymphocytes, as they can keep spontaneous metastases in permanent dormancy. Recently, CD8+ T cells are shown to play a key role in maintaining tumor dormancy, as tissue-resident memory CD8+ T cells (epidermal CD69+ CD103+ TRM cells) are implicated in the melanoma-immune equilibrium and control the active disease as demonstrated in skin-implanted melanoma cancer cells in mice model [107]. Paradoxically, while expression of B7-H1 on the surface of tumor cells impairs dormant cancer cell-killing potential of T-cells, expression of same surface receptors by natural killer (NK) cells (induced by CXCL10) can activate them to prompt a cytotoxic T lymphocyte (CTL) response resulting in destruction of otherwise CTL-killing resistant dormant tumor cells in vitro and in vivo [108]. 

Meanwhile, an association of human Tregs, neutrophils and inflammatory mediators such as interferon-gamma (IFNγ) are rather context-dependent [109]. With regards to the role of other interferons, a recent study demonstrated the role of IFN-β-induced immunological dormancy reflected in sustained activation of the IRF7/IFN-β/IFNAR axis in chemo-resistant (high dose doxorubicin and methotrexate) ER-negative 4T1 breast cancer cells in vitro and in vivo [110]. Meanwhile, one study to recapitulate sustained inflammation in lungs as demonstrated in smoker individuals revealed that neutrophil extracellular traps (NETs) proteases (elastase and MMP9) produced during inflammation can degrade laminin which further induces proliferation of dormant cells through activating αvβ3 integrin signaling, resulting in aggressive metastatic tumors in mice [111]. Immunotherapeutics that target disseminated cancer dormancy may aid to control or eliminate cancer state. In this view, treatment modalities that induce or amplify the CTL immune response or abrogate CTL immunosuppression mediated by cancer cells might be beneficial to confine or eradicate metastatic cells [112].

Dormancy state induced by the elimination of primary tumor can be avoided by a “recombinant T cell receptor ligand therapy”, dosing days in advance to tumor resection and lasting throughout the whole treatment course, that is a highly speculative therapy [113]. Alternatively, immune-stimulated dormancy pathway can be tuned to reverse dormancy and favor dormant cell eradication. For instance, blocking IDO-kynurenine-AhR metabolic circuitry abolishes immunologic latency mediated by IFN-γ in tumor-repopulating cells (TRCs). Hypothetically, the IFN-γ-STAT1 signaling induces apoptosis in proliferating tumor cells; however, TRCs-induced overexpression of IDO1 and AhR, shifts IFN-γ action and give rise to IDO1/AhR-induced p27 activation and inhibition of STAT1 signaling. Further, these events translate into the inhibition of tumor cell apoptosis and thus switching to dormancy program. Blockade of IDO/AhR metabolic circuitry not only nullifies IFN-γ-mediated latency but also promotes amplified tumor cell growth arrest by IFN-γ-dependent killing of TRCs in vitro and in vivo [48]. As noted before, dormant tumor cells may hinder CTL-mediated tumor lysis via overexpressing B7-H1 and B7.1 [42]. They also counteract apoptosis by paracrine secretion of cytokines (e.g., IL-3) and simultaneous inactivation of SOCS1, the expression of which negatively controls JAK/STAT signaling [114]. In this context, using BCR/ABL DA1-3b mouse model of acute myelogenous leukemia as a model of sustained tumor immunological dormancy, results indicated that demethylation or gene transfer restored the expression of SOCS1, which further rendered dormant cells sensitive to apoptosis and abrogated resistance to CTL-induced tumor cell destruction. Moreover, the cross-resistance to apoptosis emanating from dormant tumor cell-induced overproduction of Interleukin 3 (IL-3) was also upturned using anti-IL-3 antibody [47].

Alternatively, “recombinant T cell receptor ligand therapy” of overt cancer may retain all undetectable DTCs quiescent providing that the treatment is continued as a plan to control cancer [113]. Thus, preventing metastatic expansion would be more feasible than eliminating an established metastasis [113]. As such, tumor-cell immunization prompts tumor cell latency in mice bearing B-cell aggressive leukemia/lymphoma (BCL1). In this line, while an anti-idiotypic immunity was insufficient to eliminate BCL1 cells, it was capable of suppressing growth activating signals, which further promoted cell cycle arrest, apoptosis and persistent BCL1 dormancy in mice [115]. Also, immunity to BCL1, which was achieved by numerous inoculations of irradiated carcinoma cells, precludes leukemia growth in primary and adoptive transfer recipients even with the lifelong perseverance of residual cancer cells [116].

From a different angle, increasing inflammation instigates tumor recurrence, thus interventions that inhibit inflammatory signaling could inhibit cancer relapse [5]. In this sense, a literature search was reported that inflammation and wound healing process following surgery is enough to induce distant cancer outgrowth in cancerous patients [117], especially when the tumor outgrowth was attenuated by the adaptive immune system [118]. Accordingly, anti-inflammatory medications can benefit cancer patients undergoing surgery by avoiding the awakening of latent micrometastatic cells. Furthermore, it was shown that the lipopolysaccharide treatment-induced localized inflammation in the lungs of mice sends a wake-up signal to metastatic latency in the lung parenchyma through Zeb1 expression, a key regulator of the epithelial-to-mesenchymal transition (EMT). Likewise, Zeb1-orchestrated stimulation of EMT program by itself is sufficient to provoke metastatic spread by triggering the stable entry of tumor cells into a state of metastasis-initiating cells [45]. Accordingly, inhibition of Zeb1, LIFR: STAT3 signaling [52] as well as blocking the actions of inflammatory cytokines such as tumor necrosis factor α (TNFα) and IL-1β cytokines could confer dormancy and thus eliminate cancer outgrowth [46].

## 4. Metabolic Reprogramming

Impaired vascular system observed in solid tumors encourages establishing oxygen and nutrients deprived microenvironments that harbor metabolically stressed slow-growing cells. Tumor cells under hypoxia and nutrient deprivation become dormant by reducing or shifting their metabolic needs. A second deprivation assault would shut down the possible energy compensatory options for tumor cells and succumb them to apoptotic death. Such that, small molecule VLX600 displays superior cytotoxic action in nutrient-starved environments in vitro and in vivo and is preferentially active against quiescent cells as validated in HCT116 colon cancer spheroids. The anti-tumor potential is correlated with dampened mitochondrial respiration, culminating in bioenergetic catastrophe and tumor cell apoptosis [11]. Likewise, another study has shown the superior performance of FF-10502, a pyrimidine nucleoside antimetabolite over gemcitabine on pancreatic dormant cells in vitro and in vivo, by inducing dormant cell injury in chemotherapy-resistant cells through blocking DNA polymerase β activity and DNA repair [119]. Further, in vivo and organoid cultures to recapitulate metabolic dormancy models revealed the involvement of metabolic shifts in breast cancer relapse. That, blockade of either transportation of fatty acid into mitochondria or synthesis of cellular fatty acid lessens DNA damages and cellular reactive oxygen species (ROS) levels, connecting these hallmarks to lipid metabolism. Tumor relapse can be prevented by direct disruption of these features, either by mitigating the expansion of the residual breast cell population or scavenging ROS [50]. These metabolic changes were also reflected in transcriptomics and histological signatures of residual cancer cells from neoadjuvant-treated breast cancer patients.

An additional strategy would be reinforcing a quiescence-like metabolic state to block tumor evasion. In this regard, a novel metabolic tumor suppressor, LACTB is shown to reduce mitochondrial phosphatidylserine decarboxylase (PISD) protein abundance and phosphatidylethanolamine (PE)/lysophosphatidylethanolamines (LPE) production, resulting in a mitochondrial state consisted of reduced proliferation, increased epithelial phenotype, and a decrease in mesenchymal and cancer stem cell markers, which associated with tumor regression and inhibition of tumor formation, as demonstrated in in vitro and in vivo breast cancer models in mice and humans. Conversely, LACTB silencing in non-tumorigenic breast cell lines cooperated with oncogenic drivers (HRASG12V and MYCT58A) supporting tumor formation in xenotransplants [49]. Notably, results from a recent study showed the role of autophagy as an important regulator of metabolic tumor cell dormancy. That, the lack of autophagy, which is exploited as an important compensatory mechanism by tumor cells to provide their energy requirements, associates with an early breast cancer recurrence and escape from dormancy as demonstrated in neu-overexpressing mouse mammary carcinoma (MMC) [120].

## 5. ECM Remodeling

**Figure 2:**
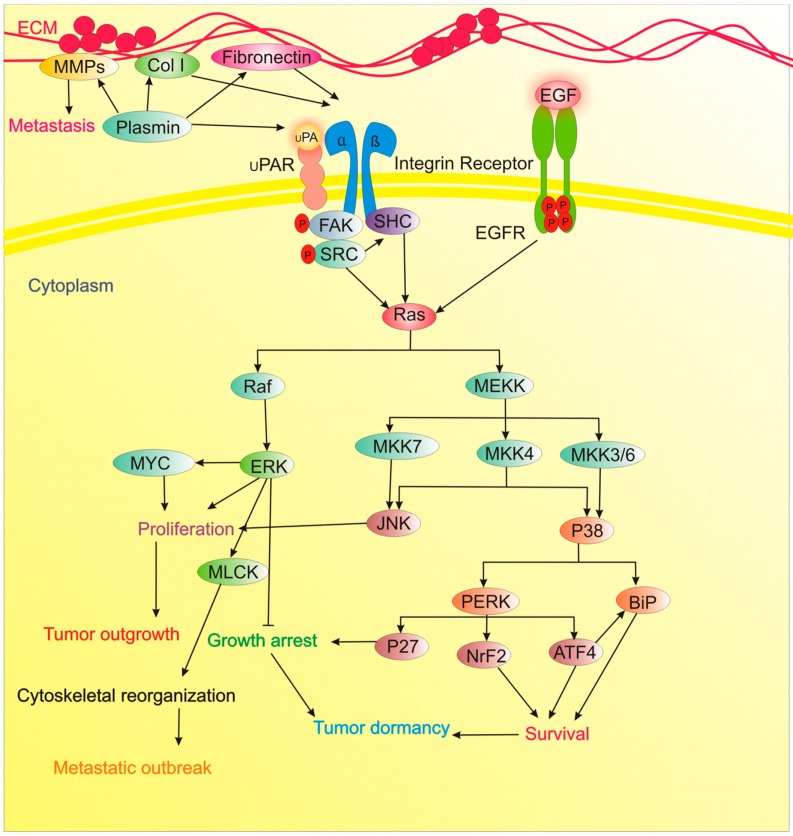
The implication of ECM and p38 signaling in tumor dormancy.

The likely involvement of communications between ECM and tumor cells in metastatic niches determine tumor latency against the metastatic outbreak. Generally, the inability of cancer cells to appropriately adhere to the ECM may potentiate their entry into a dormancy state [121]. Inhibition of one of the components of fibronectin/uPAR/integrin/ERK/MLCK signaling axis as well as suppressing PI3K/Akt pathway is sufficient to prevent/treat metastatic tumor outgrowth, meanwhile promotes dormancy state through favoring the p38 MAPK signaling [122]. As proof of concept, the switch from dormancy to the proliferating state of D2A1 cells was shown to rely on the production of Fibronectin and β1 Integrin signaling, the formation of filamentous actin (F-actin) stress fiber and cytoskeletal reorganization. In this report, integrin β1-dependent phosphorylation of myosin light chain (MLC) by MLC kinase (MLCK) was a prerequisite for F-actin stress fiber formation and exponentially growing of cancer cells. Further, blockade of β1 integrin or MLCK favored dormancy state and MLCK inhibition significantly reduced metastatic expansion in vivo [56]. Similarly, deposition of type I collagen (Col-I) to induce fibrosis is shown to induce the transition of dormant D2.0R cells to proliferating cells by β1 Integrin-mediated Src and focal adhesion kinase (FAK) activation, causing extracellular signal-regulated kinase (ERK)-dependent phosphorylation of MLC by MLCK and formation of actin stress fiber. Blockade of β1-integrin, MLCK, ERK, Src counteracted Col-I-mediated induction of this signaling cascade, cytoskeletal rearrangement, and proliferating state in the faithfully stimulated metastatic microenvironment in vivo [57]. Recently, targeting the perivascular niche with integrin inhibitors are exploited for chemosensitization of breast cancer cells and the prevention of bone metastasis in mice [123]. Likewise, Src knockdown or pharmacological blocking of SFK signaling was shown to promote p27 localization to the nucleus and thwarts transition of quiescent breast cancer cells (BCCs) into the proliferative and metastatic outbreak; still, SFK inhibition was not enough to eradicate residual cells. ERK1/2 activation was also needed for proliferation and awaking dormant cell. The combined therapy of cells undergoing the transition from dormancy to proliferating state with the MEK1/2 inhibitor (AZD6244) and Src inhibitor (AZD0530) potentiated apoptotic death in a large population of the latent cells and postponed the development of the disseminated disease, none of these was achieved with single-agent therapy. These results were obtained using the fibrosis-induced dormant-to-proliferative switch in vitro, in vivo and also ex vivo lung metastasis assay [59]. Meanwhile, EGFR signaling via activation of PI3K/AKT/mTOR and Ras/Raf/ERK axis is another contribution to tumor proliferation [124]. Conversely, TGFβ signaling represents a therapeutic opportunity to activate dormancy (Table 1, Figure 2) [67].

In the context of ECM modulation, an additional therapeutic approach can be envisioned by preventing cancer cell dormancy. For example, a valuable study reported that proliferating and dormant BCCs home in distinct areas within BM niche, with dormant BCCs, mainly occupy perisinusoidal vascular regions that are rich in stromal cell-derived factor 1 (SDF-1) and E-selectin. SDF-1 and E-selectin coordinate opposing functions in BCC, where the SDF-1/CXCR4 axis facilitates adherence of BCCs to the vascular niche, and E-selectin permits BCC entrance into the BM where they remain dormant. Thus, a combined therapy involving CXCR4/E-selectin inhibition will push cells out of their protective niches and aid trapping of the cells in the vasculature, where they could be destroyed with chemotherapy, thus provide an opportunity to control recurrent disease [68]. These observations were monitored by real-time in vivo microscopy of BM in a breast cancer xenograft model.

## 6. Cancer Stem Cells (CSCs)

DTCs or surviving tumor cells during therapy course may or may not be stem cells, in either case, however, stemness is inherent to dormancy phenotype [125]. Cumulative evidence suggests that CSCs are indeed metastasis-initiating cells, or metastatic cells acquire CSC-like phenotype upon infiltration into target organs [16,126]. Tumor cell entrance into and out of dormancy is regulated by contextual cues and intrinsic programs, like those that control the self-renewal ability of mature stem cells [125]. Furthermore, a specialized ECM niche nurse reactivation-undergoing metastatic cells, by supporting positive cues, such as Notch and Wnt, and attenuating negative cues, such as BMP [127]. Adopting a dormant state, CSCs not only can evade therapeutic killing, but the likely reversibility of this situation also poses the real threat, which can potentiate deadly relapse or recurrence decades later [128]. Notably, CSCs can adjust the expression of different surface markers which help them to colonize their desired target organs, in particular, the bone [129]. For instance, expression of chemokine receptor CXCR4 (SDF-1 receptor) by BCCs facilitates bone metastasis where osteoblasts express high levels of SDF-1 [130]. Moreover, the CXCR4/SDF-1 axis not only derives EMT for bone metastasis, but it also promotes breast cancer cell stemness, plasticity and maintenance of CSC-like properties in vivo [68]. Additional CSC-related mechanisms related to cancer cell dormancy are listed in Table 1.

## 7. Epigenetic Modification

As another key regulator of tumor dormancy, epigenetic modifications are involved in the epithelial to mesenchyme transition (EMT) which are associated with CSC phenotype and emergence of drug resistance, tumor dissemination, and a high risk of disease relapse. Identifying genes that encode these reversible alterations is an appealing therapeutic plan to combat metastatic disease by inducing differentiation of the mesenchymal cell into an epithelial phenotype [80].

In this respect, epigenetic upregulation of orphan nuclear receptor NR2F1 is detected in DTCs from the breast [131], and prostate cancer patients harboring lifelong dormant disease and in experimental latency models of head and neck squamous cell carcinoma (HNSCC). The exploitation of these epigenetic dormancy-recapitulating models revealed that NR2F1-induced dormancy depends on RARβ, SOX9 and CDK inhibitors. Also, NR2F1 induces pluripotency gene NANOG, and global chromatin repression, favoring dormancy of DTCs in the BM [82].

Likewise, polycomb-like proteins 1-3 (PCL1-3) are substoichiometric modules of the polycomb-repressive complex 2 (PRC2) that are indispensable for complex association with chromatin. Their functional redundancy is due to their opposing roles in the positive and negative regulation of cellular proliferation. Such that, in quiescent cells, expression of PCL1, a p53 target gene is predominant, while proliferating cells express E2F-regulated genes PCL2 and PCL3. Ectopic expression of any PCL protein employs PRC2 to suppress the INK4A gene; nevertheless, only PCL2 and PCL3 render an INK4A-evolved proliferative benefit. Of note, PCL1 confers a PRC2 function which acts independent of chromatin, possessing anti-proliferating effect and induce cellular dormancy through binding to and stabilizing p53 [81]. These results were attained using purified PCL expressing cell populations from the mouse hematopoietic system by flow cytometry.

Also, in vivo genome-wide short hairpin RNA screening has revealed a novel epigenetic-related mechanism involved in bone metastatic latency of estrogen receptor-positive (ER+) breast cancer. Clinical studies were reported that low levels of mitogen-and stress-activated kinase 1 (MSK1) expression links with early relapse in ER+ breast cancer patients. In this study, reduced MSK1 impaired the differentiation of breast cancer cells, and increased their bone homing and growth capacities. From a molecular perspective, MSK1 downregulation induces chromatin remodeling and decreases differentiation traits by modulating promoter chromatin status of genes encoding GATA3 and FOXA1 transcription factors and accelerates bone colonization by cells in distant micrometastatic sites [83]. Results from a recent preclinical study underpinned the role of epigenetic dormancy in patients receiving neoadjuvant letrozole treatment. Acquired resistance to estrogen depletion therapy with aromatase inhibitors was attributed to the dormancy highlighted with a global loss of DNA methylation under extended treatment [132]. Thus, epigenetic alteration and “dormancy phenotype” rather than “acquired resistance” can originate from therapy and that epigenetic markers targeting can be promising for the treatment of resistant hormone-depleted tumors.

## 8. Noncoding RNAs (miRNAs)

While the critical role of numerous microRNAs in tumorigenesis is well documented [133], the implication of dormancy miRNAs (Dmirs) is just recently illuminated [134]. For example, mesenchymal stem cell-derived exosomes comprising different miRNA contents, such as miR-222/223, are shown to induce cycling dormancy and early BC quiescent in BM and give rise to drug resistance. Further, administration of MSC-loaded antagomiR-222/223 sensitized BC cells to carboplatin-based therapy and increased survival of the mouse as the model for studying miRNA-regulated breast cancer dormancy [84]. Also, a recent study showed that metastatic outgrowth of the claudin-low mammary tumor cell line, RJ423 can be avoided by re-expression of the miR-200b/200a/429 cluster which promotes epithelial phenotype and induces tumor dormancy [87].

Moreover, a study on experimental models of fast-growing and dormant human osteosarcoma identified three dormancy-associated DmiRs regulating osteosarcoma latency: miR-200c, miR-34a, and miR-93. Accordingly, the expression of these microRNAs is lost upon the shift from avascular dormant into angiogenic fast-growing tumor state. Introduction of these miRNAs by dPG-NH2 polyplexes into MG-63 and Saos-2 cells dampened expression levels of their target genes, including hypoxia-inducible factor 1α (HIF1α), MET proto-oncogene, and moesin, vital to tumor migration and angiogenesis process. Furthermore, therapy with dPG-NH2 containing each of these microRNAs considerably extended the latency state of fast-growing osteosarcomas in mice [85].

Loss of DmiRs expression is also verified in human dormant liposarcoma, breast carcinoma, osteosarcoma, and glioblastoma tumors. In this report, transcriptional reprogramming of tumors via over-expression of DmiR-190, 588, or 580 reduced expressions of pro-angiogenic factors bFGF, TIMP-3, and TGFα while upregulated anti-angiogenic and dormancy stimulating factors angiomotin and EphA5 [135]. In all dormant tumors analyzed, overexpression of miR-190 was predominant where upregulation of this Dmir encouraged long-term dormancy of otherwise proliferative osteosarcomas and glioblastomas [17].

## 9. Stress-Induced p38 Signaling

Stress-induced activation of p38 signaling is one of the critical pathways related to tumor dormancy, which regulates a transcriptional network of 46 core genes that includes 16 transcription factors (TFs) [91]. p38 MAPK signaling coordinates the induction of growth arrest and drug-resistance in different models of cancer cell dormancy [136]. Imbalances in the activity ratio of ERK to p38 signaling is fundamental to decide the outcome of dormancy vs. tumorigenicity of different experimentally established cancer models [89].

Stress-dependent p38 activation induces dormancy by adopting a pro-survival mechanism via enhanced activation of the PERK and up-regulating the endoplasmic reticulum (ER) chaperone BiP, conferring dormant cells resistant to drug toxicity. Moreover, up-regulation of BiP suppresses activation of Bax. Thus, p38 signaling via PERK activation and BiP up-regulation secures dormant human squamous carcinoma cells (HEp3) from stress insults, such as chemotherapy in vivo [90]. Besides, the computational analysis showed that p38 activation induces the transcription of the TFs BHLHB3 and p53, while blocks FoxM1 and c-Jun expression. Also, p38-mediated activation of p53 requires down-regulation of c-Jun [91].

ATF6α as another p38-controlled transcription factor is crucial to the survival of the dormancy state. That ATF6α is vital for dormant cells adaptation to nutritional stress, chemotherapy, and, most importantly, the in vivo microenvironment. Accordingly, in dormant cancer cells, MKK6 and p38α/β control nuclear translocation and transcriptional activation of ATF6α. Downstream, ATF6α promotes survival via Akt-independent activation of mTOR signaling and up-regulation of Rheb. Thus, targeting the survival signaling axis ATF6α-Rheb-mTOR in dormant carcinoma cells may help to remove residual disease during dormancy periods [92].

As discussed before, dormant cells and active cells occupy different soils to settle in. As non-proliferative DTCs reside in the BM while metastatic growth occurs in other organs such as the lung. Accordingly, BM niche in patients serves as a metastasis “restrictive soil” by coding dormancy activating clues in DTCs. In this view, in an in vivo HNSCC carcinoma model, specific and strong TGF-β2 signaling in the BM triggered the MAPK p38α/β and resulted in low ERK/p38 signaling ratio. This favored dormancy of malignant DTCs via induction of DEC2/SHARP1 and p27, and CDK4 downregulation. Also, TGF-β2-induced latency nodes to activate SMAD1/5, TGF-β receptor-I (TGF-β-RI) and TGF-β-RIII to induce p27. In lungs, a metastasis “permissive soil” with low TGF-β2 levels, DTC latency state was transitory and continued by metastatic expansion. Importantly, systemic inhibiting of p38α/β or TGF-β-RI activities awaked dormant DTCs, and fueled multi-organ metastasis. This work reveals a “seed and soil” mechanism whereby p38α/β regulates TGF-β2 and TGF-β-RIII signaling to determine the fate of DTC dormancy and delineates permissive (lung) and restrictive (BM) microenvironments for HNSCC metastasis [93].

## 10. Conclusions and Clinical Implications

**Figure 3:**
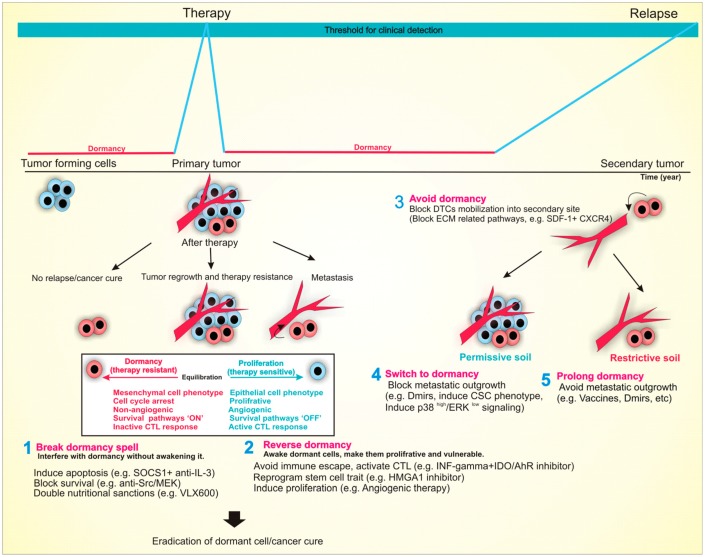
Tumor dormancy as a therapeutic opportunity to fight cancer back.

Tumor dormancy is commonly regarded as the most threatening aspect of cancer, leading to the emergence of resistance in a short time and advent of lethal metastatic outbreaks after a long latency period lasting from months to years. Now that we begin to realize dormancy tactics, several therapeutic approaches stick out to outsmart cancer. As illustrated in Figure 3, these schemes include: (i) prolonging dormant state; (ii) eradication of dormant cells; (iii) reawakening/mobilization of dormant cells and (iv) avoiding dormancy state. 

Prolonging dormant state is based upon the observation that immunotherapeutic targeting of advanced-stage cancers has extended the survival of cancer patients, yet its curative efficacy is limited due to tumor immunoediting. On the other hand, human vaccines have been able to eradicate and control many infectious diseases. The success has resulted from the administration of vaccines in prophylactic settings or during latency periods to protect an individual during future exposure to the disease rather than curing an established disease. Therefore, administration of immunotherapy at the right time is the key to success. Immunotherapeutic targeting of tumor dormancy could be more promising than targeting of advanced-stage disease to achieve a cure for cancer. Besides immunotherapy, identification of Dmirs advocates novel tools to inverse the malignant aggressive phenotype into a microscopic dormant state and may deliver encouraging targets for timely detection or prevention of cancer. Eradication of dormant cells without their awakening is the second tactic in which tricks of dormancy are leveraged against dormant cells. For example, their restrict dependence on survival pathway or altered energy metabolism could be their Achilles heel to interfere with cancer evolution and relapse. Additionally, dormant and resistant cells could be awakened by external stimuli such as growth factors and angiogenic cues, turning them into proliferative yet therapy-sensitive tumors.

Another alternative yet practical strategy would be pushing DTCs out of their protective niche (e.g., anti-CXCR4) and into a less supportive environment, where they can be targeted and eradicated by anti-cancer drugs. As the final approach is avoiding dormancy in the first place by inhibiting pathways leading to the acquisition of dormancy phenotype. However, due to the nature of some cancer cells and their escape from primary tumors at early stages before detection (they are as little as 5 mm in size), it is nearly impossible to prevent dormancy in all instances. Although most of our understanding arises from the preclinical studies, and implementation of the above-mentioned dormancy-based anti-cancer approaches remain to be weighted in the human cancer biology systems, they are reports for identifying and targeting dormant cancer cells in the clinical settings. To this, using disseminated tumor cells (DTCs) as “keyword” to search in clinicaltrials.gov, they were several clinical trials targeting DTCs in breast and prostate cancer patients. All of these trials were attempted to reduce the reservoir of DTCs in the BM to reduce cancer relapse and increase metastasis-free survival in patients. A detailed description of these trials can be found in Table 2.

Even if we can control cancer dormancy, not all cancer patients will ultimately benefit from this strategy. That is, even the clinically undetectable primary tumor could cause death in a portion of patients along with highly aggressive and metastatic tumors in the rest of patients. Although a considerable portion of disseminated dormant cells success to colonize, outgrow and become resistant; however, there is no strong evidence confirming that every single tumor cell that escape primary tumor and colonize in distant sites become resistant. 

Though the discovery of cancer cell-derived by-products such as CTCs, cell-free DNA, exosomes, and apoptotic bodies in the blood circulation and body fluids has opened the door for timely detection and non-invasive monitoring of anti-cancer therapy outcomes, given their rarity, detection of minimal residual disease in the patients is difficult, therefore, tactics to target this population of apparently asleep cells is still in its infancy. Killing may look easier in principal, but not for cancer cells; they pretend to be sleep to pretend innocence but in fact, they are just waiting for the right moment (suitable conditions, e.g., clearance of toxic chemotherapeutics) to strike back. In conclusion, as dormancy is not responsible for all metastatic deaths, targeting dormancy in such cases would be useless. Furthermore, targeting the dormant cells by induction of dormant-to-proliferative switches involves the risk of inducing metastatic progression, while drugs that target DTCs or CSCs might affect normal stem cells. At the end of the day, it is the depth of our knowledge regarding tumor dormancy schemes that will determine the fate of our fight against cancer.

## Figures and Tables

**Table 1:** Molecular cues involved in tumor cell dormancy.

Dormancy Factor	Mode	Major Findings	Ref
Angiostatin	Inducer of angiogenic dormancy	Upregulation of Angiostatin drive long-term dormancy of primary tumors, inhibit tumor growth, and reduce cancer metastases.	[31]
Thrombospondin-1	Inducer of angiogenic dormancy	Overexpression of Thrombospondin-1 inhibits melanoma angiogenesis, lung colonization, and spontaneous pulmonary metastasis.	[32]
VEGF/VPF121	Inhibitor of angiogenic dormancy	Overexpression of VEGF/VPF121 result in tumor growth and escape from dormancy.	[33]
(VEGF(121) VEGF(165) overexpression	Inhibitor of angiogenic dormancy	The level and VEGF isoforms determine the fate of aggressive tumor growth vs. nontumorigenic and dormant tumor.	[34]
VEGF(189) overexpression	Inducer of angiogenic dormancy		
Thrombospondin-1	Inducer of angiogenic dormancy	Endothelial-derived Thrombospondin-1 induces long-lasting BCC dormancy. This repressive nod is lost in sprouting neovasculature where active TGFβ1 and periostin act as tumor-promoting factors derived from endothelial tip cells.	[35]
TGFβ1, Periostin	Inhibitor of angiogenic dormancy		
Thrombospondin, Angiomotin, Tropomyosin, TGF-β2, P4HA1, EphA5, H2BK, IGFBP-5	Inducer of angiogenic dormancy	Dormant tumors undergo a stable genetic reprogramming during their switch to the fast-growing phenotype by downregulation of angiogenesis inhibitors such as Thrombospondin and decreased the sensitivity of angiogenic tumors to angiostatin along with upregulation of angiogenesis-related genes.	[36]
EGFR-1, IGF-IR, CD73, PI3K, ESM-1, PIK3CB, TIMP-3	Inhibitor of angiogenic dormancy		
MME1(NM23)	Inducer of angiogenic dormancy	NM23 inhibits EGF-induced cell migration. Increase the expression of metastasis-related genes TIMP-1, E-Cadherin and β-Catenin, reduce the expression of VEGR, CD44V6, and MMP-2 and reduce metastasis.	[37]
Kai-1 (CD82)	Inducer of angiogenic dormancy	Binding of tumor cell surface-expressed Kai1 with endothelial DARC inhibit tumor cell proliferation, induce senescence by modulating the expression of TBX2 and p21 and suppress metastasis.	[38]
BRMS1	Inducer of angiogenic dormancy	BRMS1 inhibits angiogenesis through blocking NF/KB activity. It can also reduce metastatic potential but not tumorigenicity.	[39]
HSP27	Inhibitor of angiogenic dormancy	Downregulation of HSP27 associated with reduced endothelial cell proliferation and decreased secretion of VEGF-A, VEGF-C, and induction of long-term dormancy.	[40]
CTL response, MHC class I, NK cells	Inducer of immunologic dormancy	An activate CTL response can maintain immune equilibrium with metastatic dormant cells. Immune dormancy arrest cancer cell growth and promotes angiogenic control.	[41]
B7-H1 and B7.1	Inducer of immunologic dormancy	Dormant tumor cells up-regulate B7-H1 and B7.1 and resist CTL-mediated lysis.	[42]
Macrophage (MΦs)	Inhibitor/inducer of immunologic dormancy	By forming gap junctional interactions with CSCs, the M2 MΦs promote cycling quiescence and carboplatin resistance. M1 MΦ-derived exosomes activated NFкB to reverse quiescent BCCs to cycling cells in vivo.	[43]
Neutrophils	Inhibitor of immunologic dormancy	14,15-EET trigger neutrophil infiltration in metastatic lesions by activating STAT3/JNK-hIL-8/mCXCL15 and mir-155 which converts tumor-suppressing function of neutrophils to tumor-promoting in vivo. In the presence of G-CSF/IL-6, 14,15-EET enhance STAT3 activation in neutrophils to decrease TRAIL expression and increase MMP-9 expression to induce angiogenesis during dormant micrometastases growth. Neutrophil depletion or blocking hIL-8/mCXCL15 abrogate micrometastases induced by 14,15-EET.	[44]
Zeb1	Inhibitor of immunologic dormancy	Inflammation triggers Zeb1 to promote EMT and give rise to metastatic outgrowth.	[45]
TNFα, IL-β	Inhibitor of immunologic dormancy	Addition of bone remodeling cytokines, TNFα, and IL-β to dormant cancer cells induce proliferation and occurrence of latent bone metastasis.	[46]
SOCS1, IL-3	Inducer of immunologic dormancy	T-cell inactivation and resistance to apoptosis are mediated by methylation of SOCS1, deregulation of JAK/STAT and overproduction of IL-3 by dormant cells.	[47]
IFN-γ	Inducer of immunologic dormancy	IFN-γ signaling triggers differentiated tumor cell apoptosis via STAT1; however, when IDO1 and AhR are overexpressed as in DTCs, IFN-gamma induces p27 via IDO1/AhR and inhibits STAT1 signaling, and favors dormancy state.	[48]
LPS/EGF	Inhibitor of immunologic dormancy	Activated immune/stromal cells stimulate the resident hepatic cells to derive tumor growth.	[9]
Mitochondrial dysfunction	Inhibitor of metabolic dormancy	VLX600 impairs OXPHOS and drives a HIF-1α-dependent switch to glycolysis, which this metabolic pathway can’t meet the energy demands of tumor cells, thus induction of autophagy is unavoidable. Yet, due to lack of HIF-1α-stabilization and glucose inaccessibility in metabolically stressed environments, shifting to glycolysis mode will be restricted, consequently, tumor cells undergo apoptosis.	[11]
LACTB	Inhibitor of metabolic dormancy	Mitochondrial tumor suppressor, LACTB potently inhibits the proliferation of BC cells via altering mitochondrial lipid metabolism and differentiation of BC cells by reduction of the levels of mitochondrial phosphatidylserine decarboxylase, which is involved in the synthesis of mitochondrial phosphatidylethanolamine.	[49]
FA metabolism, ROS, oxidative DNA damage	Inducer of metabolic dormancy	Residual cells display altered lipid metabolism, elevated ROS, and increased oxidative DNA damage. Thus, lipid metabolism and ROS are therapeutic targets for reducing tumor recurrence in BC patients.	[50]
NR2F1	Inducer of hypoxic dormancy	Hypoxic HNSCC and breast primary tumor microenvironments display upregulation of key dormancy (NR2F1, DEC2, p27) and hypoxia (GLUT1, HIF1α) genes. Post-hypoxic DTCs were frequently NR2F1^hi^/DEC2^hi^/p27^hi^/TGFβ2^hi^, dormant and chemotherapy-resistant.	[51]
LIFR	Inducer of hypoxic dormancy	In BC patients with bone metastases, low LIFR levels negatively correlate with HIF-1α activity and disease outcome. Hypoxia reduces the LIFR: STAT3: SOCS3 signaling in BC cells. Loss of the LIFR or STAT3 reactivates dormant BC cells to proliferate and to downregulate stem cell-related genes and specifically benefit their bone colonization.	[52]
E6/E7 antigen	Inhibitor of hypoxic dormancy	Human papillomavirus-infected cancer cells can enter into reversible dormancy state, with reducing the synthesis of viral antigen and enhanced therapeutic resistance, and uphold tumor recurrence upon reoxygenation.	[53]
Kiss-1, CRSP3	Inducer of ECM dormancy	Kiss-1 expression suppresses malignant melanoma metastasis, inhibits motility, chemotaxis, and invasion, perhaps by suppressing the expression of MMP-9. CRSP3 regulate the transcriptional expression of Kiss-1.	[54]
Type I collagen (Col-I)	Inhibitor of ECM dormancy	Atypical tetraspanin TM4SF1 as a potent inducer of metastatic recurrence of BC couples DDR1 to PKCα. This kinase activates JAK2. Then, JAK2/STAT3 activates the expression of SOX2 and NANOG, maintain the manifestation of CSC traits, and fuel metastatic recurrence in the bone, lung, and brain.	[55]
Fibronectin	Inhibitor of ECM dormancy	Fibronectin/β1 Integrin/MLCK axis induces a transition from a quiescent to proliferative, metastatic outgrowth.	[56]
Col-I	Inhibitor of ECM dormancy	Col-I/β1 Integrin/SRC/FAK/ERK/MLCK signaling induce dormant cells to switch to proliferative metastatic lesions.	[57]
u-PAR	Inducer of ECM dormancy	u-PAR, is an essential molecule in BM disseminated tumor cells for long-standing survival during dormancy by regulation of u-PAR of α5β1 integrins, and signal propagation from Fibronectin through the p38, ERK, and EGF-receptor signaling.	[58]
FAK, Src, MEK1/2 (ERK1/2)	Inhibitor of ECM dormancy	Targeting Src prevents the proliferative response of dormant cells to external stimuli. MEK1/2 inhibition suppresses their survival and eliminates tumor relapse.	[59]
KRAS/C-Myc, IGF1/AKT	Inducer of ECM dormancy	KRAS/C-Myc negative dormant cells represent an increase in autocrine IGF1/AKT. Inhibition of IGF-1R reduces residual disease burden and cancer recurrence.	[60]
TGFB2	Inducer of ECM dormancy	Cellular adhesion promotes PC cells to escape from dormancy and lethal metastasis. The mechanism involves downregulation of TGFB2, E2F4, and upregulation of MLCK, CDK6.	[61]
DIRAS family GTPase 3 (DIRAS3)	Inducer of ECM dormancy	DIRAS3 decrease ERK/AKT signaling and induce autophagy. Addition of VEGF, IGF-1, and IL-8 abrogate sustaining autophagic DIRAS3-induced dormancy. A combination of antibodies targeting VEGF, IGF-1, and IL-8 prevent outgrowth of dormant cells.	[62]
Aurora kinase A (AURKA)	Inhibitor of ECM dormancy	Activation of URKA-Erk1/2 signaling pathway induces chemotherapy resistance and promote metastasis of laryngeal squamous cell carcinoma.	[63]
Fbxw7	Inducer of ECM dormancy	Dormant breast cancer cells overexpress Fbxw7, which acts as the negative control of cell cycle and its disruption avoids entry of dormant cells into the quiescent state, rendering them sensitive to chemotherapy.	[64]
Wnt5a	Inducer of ECM dormancy	Wnt5a/ROR2/SIAH2 signaling axis is involved in the induction and maintenance of PCa cells dormancy in the bone.	[65]
TGFβ2/ GDF10	Inducer of ECM dormancy	Osteoblast-secreted proteins induce TGFβRIII-p38MAPK-pS249/T252RB pathway to mediate dormancy of metastatic PC in the bone.	[66]
Axl, Gas6	Inducer of ECM dormancy	Axl is a tyrosine kinase receptor for growth arrest-specific 6 (Gas6). Axl and Gas6 are required for TGF-β2-induced dormancy of PC cells in the bone marrow.	[67]
E-selectin, SDF-1	Inducer of ECM dormancy	Proliferating and dormant BCCs inhabit different regions, whereas E-selectin interactions allow BCC residency in the BM, the SDF-1/CXCR4 binding anchors BCCs to the metastatic niche. Blocking CXCR4 (SDF receptor) and E-selectin eliminates latent micrometastases residing in supportive bone, excising occurrence of relapsed disease.	[68]
MED12	Inducer of ECM dormancy	The lack of MED12 induces tumor cell dormancy. Re-expression of MED12 abrogates tumor cell dormancy by positively controlling EGFR expression.	[69]
N-cadherin	Inducer of CSC dormancy	N-cadherin upregulation leads to downregulation of E-cadherin, upregulation of Connexin, EMT, and dormancy.	[70]
Notch	Inducer of CSC dormancy	Notch remain activated in dormant residual cells and accelerates tumor recurrence.	[71]
CD13	Inducer of CSC dormancy	CD13 is a cancer stem cell dormancy marker in HCC.	[72]
Coco	Inhibitor of CSC dormancy	Coco enhances cancer stem cell traits and antagonizes TGF-β activity. Coco reactivates dormant BC cells in the lung whereas BMP signaling revives metastasis dormancy in the lung.	[73]
BMP7	Inducer of CSC dormancy	Bone stromal cells-derived BMP7 stimulates senescence in prostate CSCs by activating BMP7-BMPR2/p38/p21/NDRG1 axis.	[74]
SPARC	Inducer of CSC dormancy	SPARC demethylation (activation) significantly stimulate the expression of BMP7 in bone marrow stromal cells and is required for BMP7 mediated stemness and senescence of PC cells.	[75]
SOX2	Inducer of CSC dormancy	Low level of SOX2 expression is required for DTCs maintenance. SOX2 complete depletion of SOX results in activation of STAT3-p53-caspase axis and induction of cell apoptosis.	[76]
HMGA1	Inhibitor of CSC dormancy	HMGA1 reprogram triple-negative BC cells to a stem-like state, driving their metastatic outgrowth. HMGA1 silencing excise cancer stem/initiator cells and prevents oncogenesis.	[77]
TBK1	Inducer of CSC dormancy	PC cells target the HSC niche in mouse bone marrow during metastasis. Interaction with niche osteoblasts activate TBK1 expression and inhibit mTOR in PCa cells. Silencing TBK1 dampen drug resistance and formation of PCa stem-like cells.	[78]
p53, Necdin	Inducer of CSC Dormancy	Necdin-knock out adult HSCs is more proliferative and less quiescent than wild-type HSCs, indicating that Necdin resembles p53 function in supporting HSC dormancy during stable conditions.	[19]
PRRX1	Inducer of CSC Dormancy	PRRX1 positively controls dormancy through TGF-β and promoting EMT in HNSCC and its activity is correlated with low expression of miR-642b-3p and TGF-β2 and p38.	[79]
Zeb1, G9a, SMAD5, SMARCD3,	Inhibitor of Epigenetic dormancy	These genes control EMT and control dormancy by reversible activation of stem cell-like properties of breast cancer cells in vitro.	[80]
KAT5, DOT1L	Inducer of epigenetic dormancy	These genes are involved in MET, promoting epithelial morphologies and thus reduce invasive properties of breast cancer cells in vitro.	
PCL1	Inducer of epigenetic dormancy	PCL2 and PCL3 are expressed in proliferative tumor state, whereas PCL1 mainly expressed in dormant cells.	[81]
PCL2,3	Inhibitor of epigenetic dormancy		
NR2F1	Inducer of epigenetic dormancy	NR2F1 is epigenetically upregulated in tumors and induce dormancy by global chromatin repression.	[82]
MSK1	Inducer of epigenetic dormancy	MSK1 epigenetically controls the differentiation of cancer cells and its expression promotes metastatic dormancy.	[83]
miR-222/223	Inducer of Dmir dormancy	Promotes quiescence and drug resistance.	[84]
miR-34a, miR-93, miR-200c	Inducer of Dmir dormancy	Loss of DmiRNAs happens during the transition from avascular dormant into angiogenic fast-growing phenotype.	[85]
Mir16/19, miR-580, 588 or 190	Inducer of Dmir dormancy	Dmirs govern tumor dormancy, especially miR-190 induce long-lasting dormancy in glioblastomas and osteosarcomas.	[17]
miR-100-5p	Inducer of Dmir dormancy	miR-100-5p inhibition induces apoptosis in dormant PC cells and prevents the emergence of castration-resistant PC.	[86]
miR-200b/200a/429	Inducer of Dmir dormancy	Expression of these Dmirs induce tumor cell dormancy and inhibit lung metastasis of BCCs.	[87]
miR-125b	Inducer of Dmir dormancy	Its expression favors epithelial phenotype, reduces Wnt-associated stem cell signaling and mesenchymal-associated genes and thus reduce metastasis of BCCs to the bone.	[88]
p38 Signaling	Inducer of stress-induced dormancy	p38 ^Up^, ERK ^down^ leads to tumor dormancy.	[89]
	Inhibitor of stress-induced dormancy	p38^down^/ERK^up^ leads to mitogenesis.	
	Inducer of stress-induced dormancy	p38/BiP/PERK axis promotes drug resistance and survival of quiescent cells. BiP up-regulation averts Bax activation.	[90]
	Inducer of stress-induced dormancy	p38 induces dormancy by expression of p53 and BHLHB3 while inhibiting c-Jun and FoxM1.	[91]
	Inducer of stress-induced dormancy	MKK6 and p38α/β induce survival by regulating nuclear translocation and transcriptional activation of ATF6α in dormant cancer cells.	[92]
	Inducer of stress-induced dormancy	TGF-β2-MAPK p38α/β-(ERK/p38) (low)- DEC2/SHARP1, p27, ↓CDK4, and dormancy of malignant DTCs.	[93]
	Inducer of stress-induced dormancy	˧MERTK, the ↓ratio of P-Erk1/2 to P-p38, ↑ p27, NR2F1, SOX2, and NANOG, ↑histone H3K9me3 and H3K27me3, G1/G0 arrest and dormancy.	[94]
	Inducer of stress-induced dormancy	MKK4 activates MAPK, p38 and JNK, up-regulate p21 and induce cancer cell growth arrest.	[95]
	Inducer of stress-induced dormancy	MKK6 activates MAPK and p38.	[95]
	Inducer of stress-induced dormancy	MKK7 activates MAPK and JNK.	[96]

*Abbreviations*: N-myc downstream-regulated gene 1 (NDRG1); BMP receptor 2 (BMPR2); indolamine 2,3-dioxygenase 1 (IDO1)-kynurenine (Kyn)-aryl hydrocarbon receptor (AhR) (IDO1/AhR); bone morphogenetic protein 7 (BMP7); N-myc downstream-regulated gene 1 (NDRG1); secreted protein acidic and rich in cysteine (SPARC); high mobility group A1 (HMGA1); polycomb-like proteins 1-3 (PCL1-3); protein kinase-like ER kinase (PERK); urokinase plasminogen activator receptor (uPAR); breast-cancer metastasis suppressor 1 (BRMS1); mammalian target of rapamycin (mTOR); Janus-activated kinase/signal transducers and activators of transcription (JAK/STAT); suppressor of cytokine signaling (SOCS); IL-6 cytokine leukaemia inhibitory factor (LIFR); transforming growth factor β2 (TGF-β2); proline hydroxylase I (P4HA1); Eph receptor A5 (EphA5); histone H2BK; insulin-like growth factor binding protein 5 (IGFBP-5); epithelial growth factor receptor (EGFR)-1; insulin-like growth factor type I receptor (IGF-IR); 5′-Ecto-nucleotidase (CD73); endothelial cell–specific molecule 1 (ESM-1); phosphatidylinositol 3-kinase PI3K (PIK3); tissue inhibitor of metalloproteinase-3 (TIMP-3). The RNA polymerase II transcriptional mediator subunit 12 (MED12). ˧ denotes suppression, ↑ denotes upregulation, ↓ denotes downregulation.

**Table 2:** Clinical trials targeting persistent DTCs residing in the protective BM niche.

Trial Name	Phase	ClinicalTrials.gov Identifier	Anti- Dormancy Strategy	Targeting Agent	End-Point	Results
Secondary adjuvant treatment for patients with isolated tumor cells in bone marrow	II	NCT00248703	Addition of docetaxel in the adjuvant treatment to reduces the risk of persistent DTCs	docetaxel	Disease-free survival by DTC status; DTC number in BM aspirate	DTC eradication in 79% of patients; enhanced metastasis-free survival
CLEVER pilot trial: A phase II pilot trial of hydroxychloroquine, everolimus or the combination for prevention of recurrent breast cancer	II	NCT03032406	Target persistent DTCs following standard of care treatment in breast cancer patients	hydroxychloroquine, everolimus or combination	Number of adverse events; DTC number in BM aspirate	N/A
Zoledronic acid in the treatment of breast cancer with minimal residual disease in the bone marrow (MRD-1)	II	NCT00172068	Inhibitor of bone resorption; interrupt dormancy state of DTCs	zoledronic acid + calcium/vitamin D	Reduction of detected tumor cells in BM	N/A
A phase Ib/II trial of gedatolisib, hydroxychloroquine or the combination of prevention of recurrent breast cancer (GLACIER)	Ib/II	CT03400254	Target persistent DTCs following standard of care treatment in breast cancer patients	hydroxychloroquine, gedatolisib or combination	DTC number in BM aspirate	N/A
A pilot study to evaluate the impact of denosumab on disseminated tumor cells in patients with early-stage breast cancer	II	NCT01545648	Interrupt immunological dormancy by blocking RNKL overexpression by DTCs which foster the production of chronic inflammatory cytokines.	denosumab	DTC number in BM aspirate	N/A
A pilot study of mobilization and treatment of disseminated tumor cells in men with metastatic prostate cancer	I	NCT02478125	Anti-CXCR4 strategy can be used to mobilize and target persistent DTCs	burixafor hydrobromide, G-CSF, docetaxel, or combination	CTC number in peripheral blood; HSC number in peripheral blood; PSA response, safety	N/A
A pilot study of the combination of 5-azacitidine (5-AZA) and all-trans retinoic acid (ATRA) for prostate cancer (PCa) with PSA only recurrence after definitive local treatment	II	NCT03572387	Reprogramming therapy in patients with recurrent PCa based on rising PSA only.	Combination of 5-azacitidine and alltrans retinoic acid, and lupron	Disease progression-free rate, Percentage of adverse events by grade, time to tumor progression, measurement of dormancy markers TGF-β2, BMP7, BMP4, GAS6, retinoic acid and NR2F1	N/A
Effect of trastuzumab on disease-free survival in early-stage HER2-negative breast cancer patients with ERBB2 expressing disseminated tumor cells	II	NCT01779050	Targeted trastuzumab therapy to eliminate HER2 expressing disseminated tumor cells in BM.	doxorubicin, trastuzumab, cyclophosphamide, paclitaxel, epirubicin, docetaxel, carboplatin, fluorouracil, and combination	Elimination of ERBB2-positive DTCs from BM. Improved disease-free survival.	N/A
